# Sc-Modified C_3_N_4_ Nanotubes for High-Capacity Hydrogen Storage: A Theoretical Prediction

**DOI:** 10.3390/molecules29091966

**Published:** 2024-04-25

**Authors:** Shuli Liu, Xiao Tang, Chang He, Tingting Wang, Liying Shang, Mengyuan Wang, Shenbo Yang, Zhenjie Tang, Lin Ju

**Affiliations:** 1 School of Physics and Electric Engineering, Anyang Normal University, Anyang 455000, China; 01958@aynu.edu.cn (S.L.); 211104005@stu.aynu.edu.cn (C.H.); 211104029@stu.aynu.edu.cn (T.W.); 211104023@stu.aynu.edu.cn (L.S.); 211104027@stu.aynu.edu.cn (M.W.); zjtang@aynu.edu.cn (Z.T.); 2 College of Science, Institute of Materials Physics and Chemistry, Nanjing Forestry University, Nanjing 210037, China; xiaotang@njfu.edu.cn; 3 Hongzhiwei Technology (Shanghai) Co., Ltd., 1599 Xinjinqiao Road, Pudong, Shanghai 201206, China; yangshenbo@hzwtech.com

**Keywords:** C_3_N_4_ nanotube, hydrogen storage, density functional theory calculations, Sc modification

## Abstract

Utilizing hydrogen as a viable substitute for fossil fuels requires the exploration of hydrogen storage materials with high capacity, high quality, and effective reversibility at room temperature. In this study, the stability and capacity for hydrogen storage in the Sc-modified C_3_N_4_ nanotube are thoroughly examined through the application of density functional theory (DFT). Our finding indicates that a strong coupling between the Sc-3d orbitals and N-2p orbitals stabilizes the Sc-modified C_3_N_4_ nanotube at a high temperature (500 K), and the high migration barrier (5.10 eV) between adjacent Sc atoms prevents the creation of metal clusters. Particularly, it has been found that each Sc-modified C_3_N_4_ nanotube is capable of adsorbing up to nine H_2_ molecules, and the gravimetric hydrogen storage density is calculated to be 7.29 wt%. It reveals an average adsorption energy of −0.20 eV, with an estimated average desorption temperature of 258 K. This shows that a Sc-modified C_3_N_4_ nanotube can store hydrogen at low temperatures and harness it at room temperature, which will reduce energy consumption and protect the system from high desorption temperatures. Moreover, charge donation and reverse transfer from the Sc-3d orbital to the H-1s orbital suggest the presence of the Kubas effect between the Sc-modified C_3_N_4_ nanotube and H_2_ molecules. We draw the conclusion that a Sc-modified C_3_N_4_ nanotube exhibits exceptional potential as a stable and efficient hydrogen storage substrate.

## 1. Introduction

The growing developments of human communities lead to an ever-increasing demand for fossil sources of energy. This phenomenon will lead to over-exploitation and scarcity of fossil energy. Meanwhile, hazardous gases produced by burning fossil energy pollute the environment [1,2], and the released carbon dioxide also results in global warming, causing the greenhouse effect [3,4,5,6], leading to a rise in sea levels and other adverse effects [7]. Finally, the population’s quality of life is significantly reduced due to damage to Earth’s ecosystem. Therefore, finding an environmentally friendly alternative to solve the energy problem is urgently required [8,9,10,11,12]. With its plentiful reservoirs, high specific energy, good combustion characteristics, non-toxicity, non-hazardous, and non-polluting qualities, hydrogen is an extremely effective and clean energy source. Although creating hydrogen from decomposing water has advanced significantly in recent years, locating appropriate storage materials is still a major challenge [13,14,15,16]. Extensive studies on the characteristics of liquid and solid hydrogen storage materials have been initiated in an attempt to address this issue. Solid hydrogen storage materials are often a superior option, given the high energy consumption of hydrogen liquefaction and the high requirements of hydrogen storage containers. In addition, achieving a high weight density and reversibility in terms of hydrogen storage materials is also crucial at room temperature [17,18,19]. Based on the US Department of Energy (US-DOE) standard [20,21], substrates are required to possess remarkable capacities for storing hydrogen while also displaying adsorption energy ranging from −0.2 eV to −0.7 eV for H_2_ molecules [22]. In addition, hydrogen storage capacity density requires being above 6.5 wt%. To identify the optimal substrate for hydrogen storage, researchers have examined various materials, including metal hydrides [23,24,25], metal alloys [13,26,27,28], metal–organic frameworks [14,29], and zeolites [30]. Yu et al. discovered that metal alloys were difficult to dehydrogenate in hydrogen storage applications and require a higher temperature for dehydrogenation [13]. Sakintuna et al. reported that magnesium-based cyanide held a reversible hydrogen storage capability of up to 7.6 wt%, but its hydrogen resolution temperature is as high as 300 degrees Celsius [25]. The difficulties encountered by these materials are high desorption temperatures and structural instability at high temperatures. Therefore, the development of materials that possess reversible hydrogen storage properties at ambient temperature is urgently needed.

Porous systems are considered viable hydrogen storage alternatives due to their good reversibility, mild hydrogen absorption/desorption conditions, and other advantages [31,32,33]. As a new type of porous 2D material, C_3_N_4_ exhibits excellent hydrogen storage characteristics relating to its large porous structure, lightweight, good thermal stability, and enviable surface-to-volume ratio; therefore, it has garnered a lot of attention [34]. Previously, Chakraborty [35] et al. demonstrated the potential hydrogen storage capabilities of flat C_3_N_4_ [36]. However, the van der Waals (vdW) interaction between H_2_ molecules and the pure layered nanostructures is weak, leading to low hydrogen storage capacity at room temperature. Doping or metal-modifying nanostructures are methods of improving hydrogen adsorption because the modification of metals enhances the surface activity of porous materials and further promotes their interaction with H_2_. Some studies [37] have demonstrated that alkali metal doping may improve the hydrogen absorption of carbon nanotubes. Li-functionalized C_3_N_4_ increased its hydrogen storage capacity to 10 wt%., as demonstrated by Wu et al. Notably, at 0 K, Li and C_3_N_4_ have a low binding energy. The low binding energy would cause Li clusters to develop at a higher temperature, which would reduce the capacity of the hydrogen storage system [38]. As opposed to alkali metal elements, transition metal (TM) elements present more diverse and malleable outer electronic structures that allow them to interact with numerous substances and can alter bonding energy. Recent studies and simulations have also demonstrated that TM atoms or ions may bind hydrogen molecules and present optimal binding energies for practical uses [39,40,41,42,43]. Studies conducted by E. Durgun and colleagues demonstrated that a carbon atomic chain with two titanium atoms added may obtain a 14.4 wt% hydrogen storage capacity, greatly boosting the hydrogen storage mass density of carbon nanotubes [44]. Nachimuthu et al. showed that boron-doped graphene modified with TM atoms was a viable candidate material for improving reversible hydrogen storage capacity [45]. Sun et al. found that metal clusters formed by titanium atoms on carbon nanotubes altered the hydrogen binding and reduced the weight density percentage of hydrogen storage [46]. Sathe et al. found that Ti-modified C_24_ fullerene can adsorb 4 H_2_ with 10.5 wt% hydrogen storage; however, the ultra-high desorption temperature is a severe problem [45]. Different from other TM atoms, scandium, being the lightest TM, has an abundance of vacant 3D orbitals, which are conducive to the formation of bonds with hydrogen molecules. Sc-doped C_60_ fullerene structures have shown that scandium stands out as one of the most optimal transition metal dopants for enhancing hydrogen storage in fullerene molecules. Vikram et al. also experimentally demonstrated Sc-modified carbon nanostructures as potential candidates for hydrogen storage [36]. Inspired by these reports, scandium modifications to C_3_N_4_ nanotubes are expected to improve their hydrogen storage performance.

Our study shows that Sc-modified C_3_N_4_ nanotubes have a strong hydrogen storage capacity and have the benefit of releasing hydrogen gas at room temperature. Based on the simulation, Sc-modified C_3_N_4_ nanotubes show better stability even at high temperatures, which makes it harder for the Sc atom to break free from C_3_N_4_ nanotubes. Moreover, a strong migration barrier keeps Sc atoms stable inside the macrocycle and inhibits the creation of metal clusters. First-principles density-functional theory simulations demonstrate that H_2_ has a higher probability of diffusing along the tube; Sc-modified C_3_N_4_ nanotubes have the capacity to adsorb up to nine hydrogen molecules; their hydrogen storage mass density can reach 7.287 wt%; and the average binding energy is −0.20 eV/H_2_, which is compliant with US Department of Energy regulations. Charge density and Bader charge analyses were performed to investigate the adsorption mechanism of Sc-modified C_3_N_4_ nanotubes in terms of absorbing H_2_ molecules. The results show that there is a weak van der Waals connection and Kubas interaction between Sc atoms and H_2_ gas. Sc-modified C_3_N_4_ nanotubes had an average desorption temperature of 258 K, indicating that the structure may desorb the H_2_ at −15 °C without the need for further energy. As a result, Sc-modified C_3_N_4_ nanotubes perform very well as a hydrogen storage medium. Our findings may provide a path to developing a novel, highly effective hydrogen storage material that can utilize hydrogen at normal temperatures and store it at low temperatures.

## 2. Results and Discussion

### 2.1. Geometric Structures of Pure C_3_N_4_ Nanotube and Single H_2_ Molecule Adsorption

**Figure 1:**
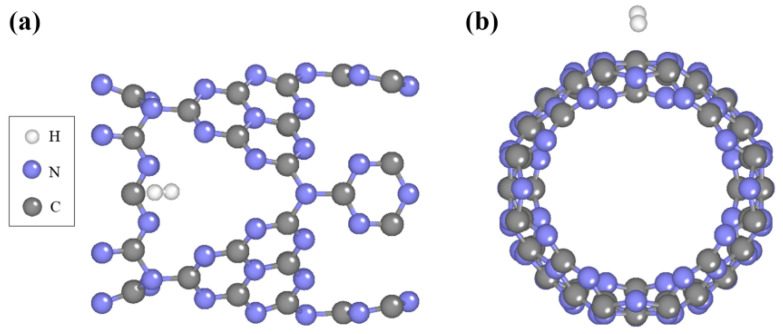
The figure shows the (**a**) top and (**b**) side views of the most stable adsorption systems of H_2_ on the surface of a pure C_3_N_4_ nanotube.

We first investigated the geometric structures of C_3_N_4_ nanotubes and H_2_ molecule adsorption properties. As shown in Figure S1, C_3_N_4_ nanotubes possess rich pore structures with a large specific surface area, which is favorable for exposing more active sites, effectively improving hydrogen storage capacity. Based on its structural characteristics, it can be found that there exist eight potential locations for the adsorption of hydrogen on pure C_3_N_4_ nanotubes. This includes k1 and k3 (above the C atom in the pore), k2 and k4 (above the N atom in the pore), k5 and k6 (above the hexagonal ring), and k7 and k8 (above the two adjacent and next-to-next adjacent N atoms in the macrocycle). Therefore, we studied the adsorption of H_2_ at different sites of H_2_ at different sites of pure C_3_N_4_ nanotubes. According to Formula (1), the adsorption energy of a H_2_ molecule on pure C_3_N_4_ is calculated to measure the change in energy during adsorption. The adsorption energy of hydrogen at different adsorption sites is shown in Figure S2. Due to the lowest adsorption energy, k8 is the most possible adsorption site for H_2_ molecules, and the corresponding relaxed configuration for the adsorption system is shown in Figure 1. The H_2_ molecule is about 3 Å away from the surface of the nanotube, indicating weak physisorption. Moreover, the adsorption energy of all sites considered for the pure C_3_N_4_ nanotube does not satisfy the range (DOE-US) of −0.2 eV~−0.7 eV. In other words, pure C_3_N_4_ nanotubes are not suitable for hydrogen storage materials because the adsorption energy of H_2_ on pure C_3_N_4_ nanotubes is too unstable. 

### 2.2. Structure and Stability of Sc-Modified C_3_N_4_ Nanotubes

**Figure 2:**
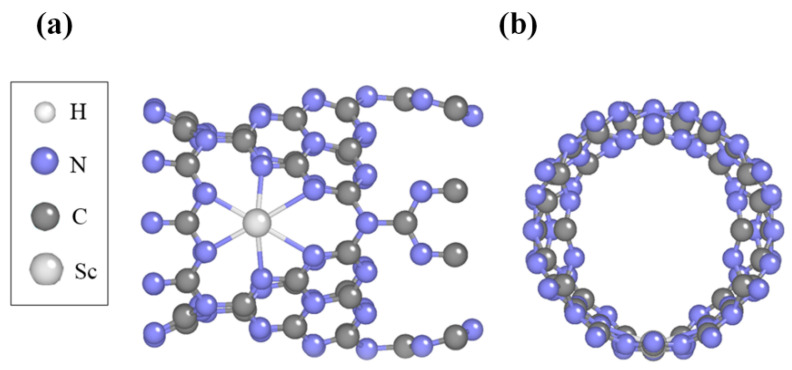
The figure shows the (**a**) top and (**b**) side views of the optimal configuration of a Sc-modified C_3_N_4_ nanotube.

Modifications to C_3_N_4_ materials using Sc metal are expected to improve the stability of H_2_ molecule adsorption. The locations at which Sc atoms are incorporated can influence the structural stability and the efficacy of hydrogen storage in C_3_N_4_ nanotubes. Considering the potential locations of H_2_ adsorption in Figure S1, we studied modifying k1–k8 of pure C_3_N_4_ using Sc atoms to obtain a stable structure. After structural optimization, their bonding energies were calculated with Formula (2), and the results are listed in Table S1. A negative bonding energy between isolated Sc atoms and pure C_3_N_4_ nanotubes indicates an exothermic reaction, which suggests structural stability. The optimized structures of Sc-modified C_3_N_4_ nanotubes at the k7 and k8 sites tend to end up in the same configuration, and we found that both of the sites present the smallest bonding energy when comparing all of the obtained bonding energies of Sc atoms at different desorption sites in C_3_N_4_ nanotubes. Therefore, the macrocycle’s core is the most stable location for metal Sc atoms to adsorb in nanotubes. As shown in Figure 2, every macrocycle favoring a single Sc atom when Sc atoms are modified on C_3_N_4_ could avoid the clustering issue for Sc decoration in C_3_N_4_ nanotubes. An analysis of the elastic modulus reveals that single-atom Sc decoration has a minimal impact on the mechanical properties (a bit softer) of the C_3_N_4_ nanotubes. For further details, refer to the Supplementary Materials and Figure S3.

**Figure 3:**
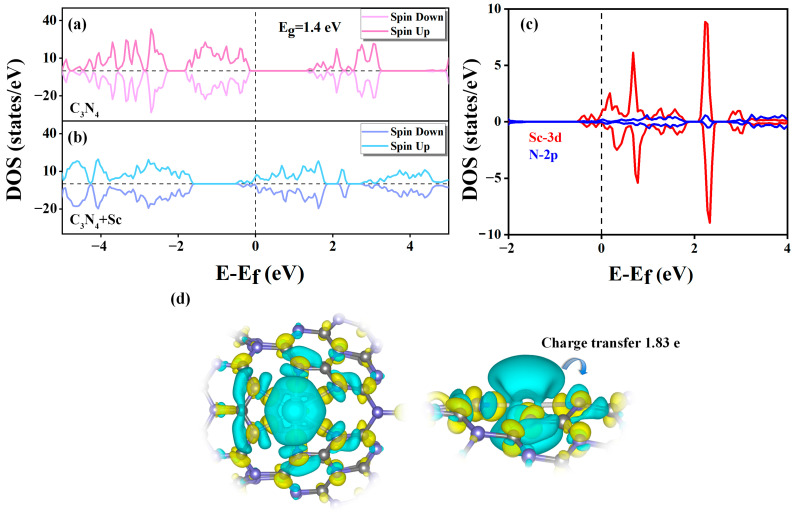
The figure shows the DOS of (**a**) pure C_3_N_4_ nanotube and (**b**) Sc-modified C_3_N_4_ nanotube. (**c**) PDOS for Sc-3d orbitals and N-2p orbitals in Sc-modified C_3_N_4_ nanotubes. Fermi level is set at 0 eV. (**d**) The CDD of Sc-modified C_3_N_4_ system with the isosurface value of 0.003 e/Å^3^. Cyan and yellow regions separately represent the electron-rich and electron-deficient regions.

The interplay between Sc atoms and C_3_N_4_ nanotubes was examined using the total density of states (DOS). Figure 3a demonstrates that the DOS of pure C_3_N_4_ nanotubes exhibit symmetry between the up and down spins due to their nonmagnetic behavior (μ = 0 μB). However, in Figure 3b, the introduction of Sc atoms into C_3_N_4_ nanotubes breaks the symmetry of the up and down spins, resulting in spin polarization and the emergence of a magnetic moment in the Sc-modified C_3_N_4_. The band gap of the pure C_3_N_4_ nanotube is calculated to be 1.40 eV, whereas the Sc-modified C_3_N_4_ nanotube lacks a band gap and exhibits a metal-like nature. It is evident that the doping of Sc atoms in the C_3_N_4_ nanotubes alters the electronic structure of the substrate, which makes the nanotubes more metallic. It is anticipated that this might increase the hydrogen storage capacity of Sc-modified C_3_N_4_ nanotubes as they have more active electrons interacting with H_2_ molecules. To uncover the nature of orbital interactions and understand the binding process within Sc-modified C_3_N_4_ nanotubes, we have analyzed the differences in the partial density of states (PDOS) and charge density. As shown in Figure 3c, Sc doping results in numerous new hybridization peaks at the Fermi energy level for the Sc-3d and N-2p orbitals. The hybridization between N and Sc atoms elucidates a strong orbital interaction between Sc atoms and C_3_N_4_ nanotubes. Additionally, an increase in the DOS can lead to the formation of chemical bonds, which forecasts the superior bonding energy and enhanced structural stability of Sc-modified C_3_N_4_ nanotubes. To further determine the precise number of electrons transferred from the Sc atoms, we conducted Bader charge analysis. The analysis revealed that each Sc atom transferred 1.83 e to the N-2p orbital of the C_3_N_4_ nanotubes. Then, as shown in Figure 3d, we plotted three-dimensional (3D) charge density difference (CDD) images. Figure 3c,d support the notion that in the bonding mechanism between Sc and C_3_N_4_ nanotubes, some electrons migrate from the 3D orbitals of single Sc atoms to the 2p orbitals of N atoms, thus forming covalent bonds. Based on the above analysis, it can be inferred that the combined interactions between Sc atoms and C_3_N_4_ nanotubes, with Sc atoms transferring 1.83 e to N atoms, form covalent bonds that can stabilize the system.

**Figure 4:**
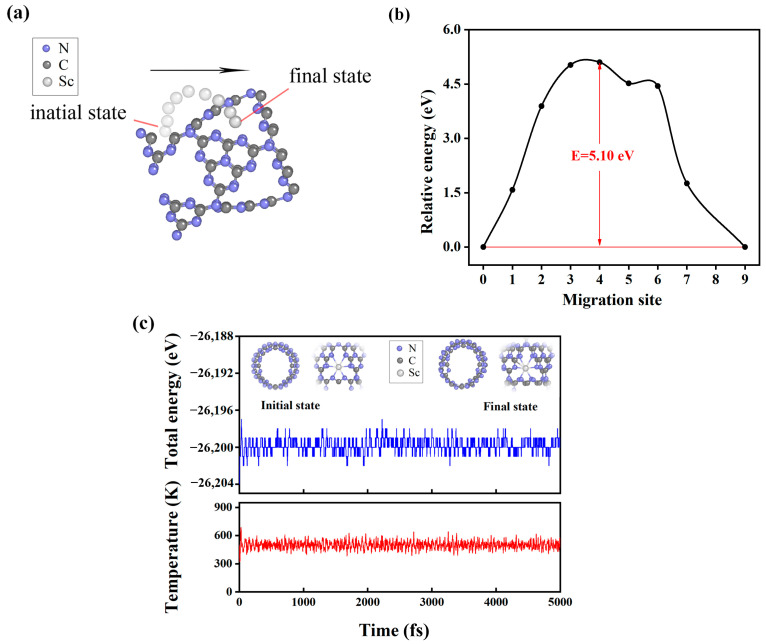
The figure shows (**a**) schematic diagram of migration path of Sc atoms from one equilibrium position to the next neighboring equilibrium position. (**b**) The corresponding diffusion energy barrier. (**c**) AIMD simulation on the change of the total energy Sc-modified C_3_N_4_ nanotubes for 5 ps with a time step of 1 fs at 500 K. The corresponding temperature over all simulation time.

The stability of a hydrogen storage system influences hydrogen storage performance; therefore, we performed diffusion energy barrier calculation to determine the stability of Sc atoms in each macrocycle, and ab initio molecule dynamics (AIMDs) simulations relating to a Sc-modified C_3_N_4_ nanotube were applied to measure the structural integrity at high desorption temperatures. The clustering of metal atoms within the system can occur readily if the transition metal atom’s diffusion energy barrier is close to its thermal energy at the highest desorption temperature. Therefore, we first calculated Sc-atom thermal energy at the peak resolved temperature of 500 K according to the following equation: E = 3/2k_B_T, where k_B_ and E represent the Boltzmann constant and the thermal energy of the Sc atom, respectively. Furthermore, the value of T was set at 500 K, which exceeds the desorption temperature. The calculated thermal energy is about 0.065 eV. Then, we moved the Sc atoms from one equilibrium position to the next neighboring equilibrium position, as shown in Figure 4a. The corresponding migration barriers of Sc atoms were calculated, and the obtained values are plotted in Figure 4b. The obtained maximum migration barrier is 5.10 eV, which greatly exceeds the aforementioned thermal energy (0.065 eV), suggesting that it takes a large amount of energy for Sc atoms to jump from the center of one macrocycle to another. The results of migration barriers calculated with other exchange-correlation functional also support this opinion. More details can be found in the Supplementary Materials. Hence, the Sc-modified C_3_N_4_ nanotubes possess high stability, i.e., the Sc atoms are not susceptible to metal agglomeration. Given that the substrate’s structural integrity during heat variations is related to the durability of the hydrogen storage substrate at high desorption temperatures. Therefore, for the real-world utilization of Sc-modified C_3_N_4_ nanotubes as hydrogen storage materials, ensuring their structural stability under high desorption temperatures is very essential. Next, we performed AIMD simulations to examine the stability of the Sc-modified C_3_N_4_ nanotube at 500 K. As displayed in Figure 4c, the total energy of the Sc-modified C_3_N_4_ system oscillates around the mean value of the 500 K simulation time data with small variations, which indicates that the system is structurally robust at high temperatures. In addition, many metal-doped g-C_3_N_4_ catalysts have been fabricated experimentally [47,48,49]. Therefore, Sc-modified C_3_N_4_ nanotubes are feasible.

### 2.3. H_2_ Molecules Adsorption on Sc-Modified C_3_N_4_ Nanotubes

**Figure 5:**
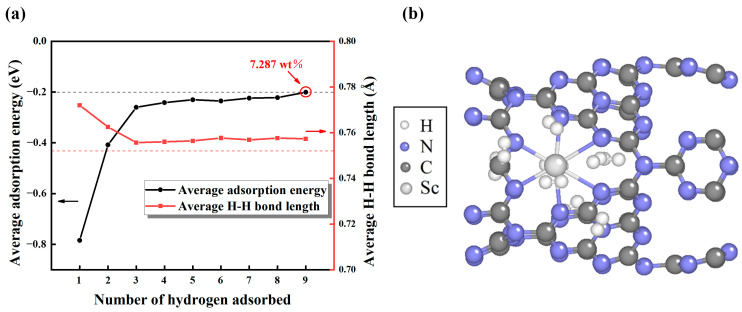
The figure shows (**a**) average H-H bond length and adsorption energy of 1–9 hydrogen adsorbed on Sc-modified C_3_N_4_ nanotubes. The black dotted line shows *E*_ads_ = −0.2 eV, and the red dotted line shows the isolated hydrogen bond length *l* = 0.752 Å. (**b**) The configuration of Sc-modified C_3_N_4_ nanotubes with nine H_2_ molecules adsorbed.

In Sc-modified C_3_N_4_ nanotubes, we discovered that hydrogen was adsorbed at the position of Sc single atom, and we calculated the average absorption energy of hydrogen molecules. First, the initial hydrogen molecule was positioned 2.487 Å away from the Sc-modified C_3_N_4_ nanotubes. After optimizing this structure, the hydrogen molecule was changed to 2.255 Å, and the calculated adsorption energy was −0.79 eV. We can conclude that the adsorption energy of the first H_2_ molecule is larger than the adsorption energies of H_2_ molecules guided by DOE-US (−0.2 eV to −0.7 eV). This suggests that the first hydrogen molecule has a stronger ability to be absorbed on Sc-modified C_3_N_4_ nanotubes. Additionally, by considering structural symmetry, we examined the potential adsorption locations for each extra hydrogen molecule. To comprehensively explore the adsorption capacity of Sc-modified C_3_N_4_ nanotubes, we added hydrogen molecules in turn (Figure S4) and calculated the changes in H-H bond length and average adsorption energy. The computational results are shown in Figure 5a. Obviously, as the count of H_2_ molecules rises, the average adsorption energy tends to diminish. When nine H_2_ molecules are added, the average adsorption energy reaches the upper limit of the DOE-US standard (−0.2 eV) [21]. This indicates that with the ongoing addition of H_2_ molecules, H_2_ molecules will inhibit interactions with the substrate and might escape into free H_2_ molecules. Hence, we determined that nine molecules may represent a likely limit for full hydrogen saturation per Sc atom. The optimized maximum adsorption configuration is shown in Figure 5b, where nine hydrogen molecules can be adsorbed near each Sc atom. As shown in Figure S5, we combined a Sc atom at the most stable position of each large ring and obtained a mass fraction of 19.7 wt% of Sc elements, according to Formula (3). Based on Formula (4), the final hydrogen storage mass density of the Sc-modified C_3_N_4_ nanotubes reached 7.29 wt%, exceeding the DOE-US standard (6.5 wt%) [21], and is superior to many other hydrogen storage systems, such as Ti-decorated boron-doped twin-graphene (4.95 wt%) [50] and Sc-decorated graphene with pyridinic-N defects (4.95 wt%) [51]. The corresponding H-H bond length increases from 0.75 Å to 0.77 Å, which is close to the isolated hydrogen bond length, confirming that H_2_ molecular stability forms after adsorbing on the nanotube. In addition, we considered the effect of defects (C and N point defects) and humid environments on hydrogen storage efficiency and stability. The calculated results demonstrate that, though the introduction of defects could enhance the stability of Sc atom decoration in C_3_N_4_ nanotubes, it brings about poor adsorption energy for the H_2_ molecule, reducing hydrogen storage efficiency and stability. The adsorption energy of H_2_ molecules increases to 0.073 eV in a humid environment. Such high adsorption energy means that this system cannot adsorb H_2_ in this case. The possible reason for this is that H_2_O molecules are passive in the active site of H_2_ adsorption and hinder the adsorption of H_2_ molecules (Figure S6). The details can be found in the Supplementary Materials.

### 2.4. Interaction between H_2_ and Sc-Modified C_3_N_4_ Nanotube

**Figure 6:**
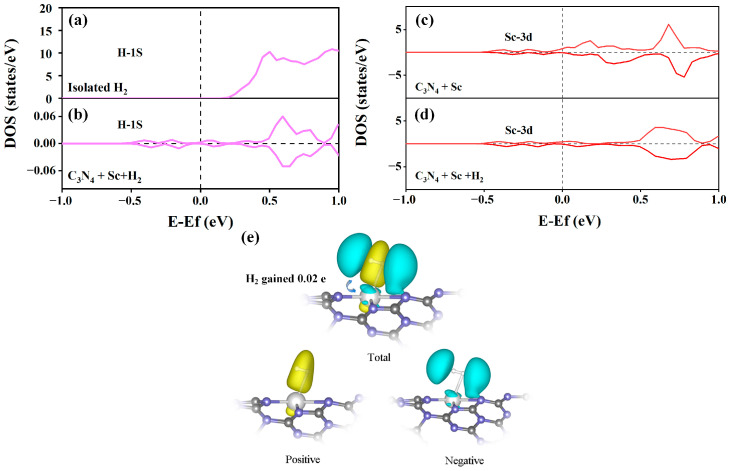
The figure shows the PDOS of the H-1s orbitals of (**a**) the isolated H_2_ molecule and (**b**) C_3_N_4_ + Sc + H_2_ system. PDOS of Sc-3d orbital of (**c**) C_3_N_4_ + Sc and (**d**) C_3_N_4_ + Sc + H_2_ systems. (**e**) The CDD of C_3_N_4_ + Sc + H_2_ system with the isosurface value of 0.003 e/Å^3^. Cyan and yellow regions represent the electron-rich and electron-deficient areas, respectively.

Figure 6 illustrates our analysis of the PDOS for H-1s and Sc-3d orbitals, which aided in understanding the mechanics of charge transfer and the interaction between the electronic orbitals of Sc-modified C_3_N_4_ nanotube and the adsorbed H_2_ molecules. In Figure 6a,b, compared with isolated hydrogen molecules, H-1s orbital eigenstates are strengthened after H_2_ is absorbed on Sc-modified C_3_N_4_ tubes, which suggests that the H-1s orbital gains charge. In addition, in Figure 6c,d, the Sc-3d orbitals eigenstates are weakened and lose charge. Hence, the improved hydrogen storage capacity of Sc-modified C_3_N_4_ nanotubes can be attributed to the charge migration from the Sc-3d orbitals to the H-1s orbitals upon the absorption of H_2_ by the Sc-modified C_3_N_4_ nanotube. As discussed above, the H-H bond length elongates slightly after the hydrogen molecule is absorbed on the Sc-modified C_3_N_4_ nanotubes. It can be speculated that this charge transfer leads to the slight elongation of the H-H bond. To confirm this conjecture, we also calculated the differential charge and plotted the charge density of three-dimensional images to analyze the charge transfer situation, as shown in Figure 6e. Observing the charge density images, we can observe that both H_2_ and Sc atoms have both charge loss regions and charge gain regions, indicating that there is both charge donation and back donation in the two atoms. When hydrogen molecules are adsorbed onto the scandium-modified C_3_N_4_ nanotube, a reverse charge transfer occurs from the scandium’s filled 3D orbitals to the hydrogen’s vacant lowest unoccupied molecular orbitals. Simultaneously, there is also a charge transfer from the hydrogen’s filled highest occupied molecular orbitals to the unoccupied 3D orbitals of scandium. The H-1s orbital gains a little net charge (0.02 e) during the processes of charge donation and back donation, which can promote orbital interactions and lengthen the H-H bond. Therefore, the Kubas interaction and weak van der Waals interactions are primarily responsible for the binding of hydrogen molecules with the scandium atom [35]. To further explain the Kubas interaction, we plotted the PDOS of the H-1s orbital from the hydrogen adsorbed versus the Sc-3d orbital from the substrate in Figure S7. At the near-Fermi energy level, the hybrid peak of the H-1s orbital and Sc-3d orbital is almost in the same energy range, which indicates that the H-1s orbitals appear to be coupled to the Sc-3d orbitals.

### 2.5. Diffusion Energy Barrier for Hydrogen in a Tube

**Figure 7:**
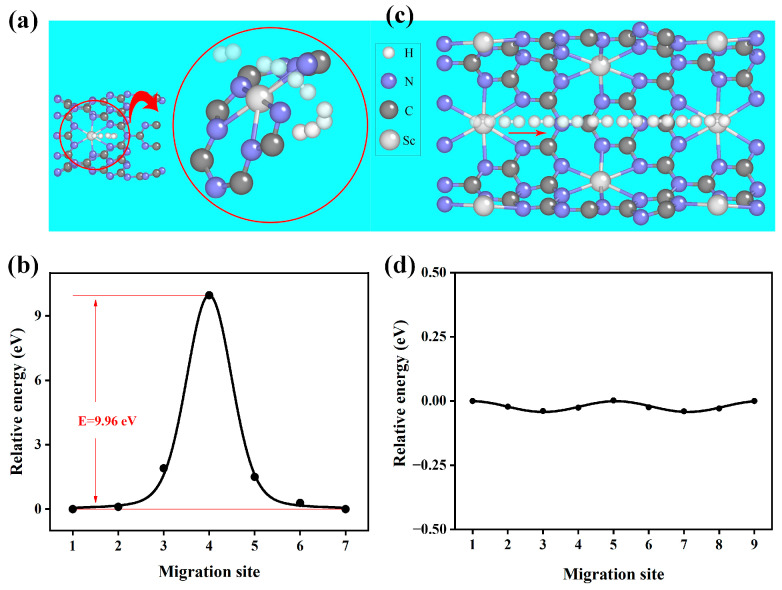
The figure shows the (**a**) schematic diagram of H_2_ diffusion in the macrocycle of C_3_N_4_ nanotubes. The arrow means the direction of the H_2_ migration. (**b**) The energy barrier for H_2_ diffusion in the macrocycle of C_3_N_4_ nanotubes. (**c**) Schematic diagram of H_2_ diffusion along the inner channel of C_3_N_4_ nanotubes. The arrow shows the direction of H_2_ diffusion. (**d**) The energy barrier of H_2_ diffusion along the channel of C_3_N_4_ nanotubes.

The distribution of adsorbed H_2_ on both sides of the nanotubes provides the basis for the hydrogen desorption capacity that was previously described. Therefore, we must figure out the probability of hydrogen getting inside the nanotube. Obviously, there are two ways that H_2_ can enter the interior: either entering the interior through the macrocycle in the side wall or via diffusion along the tube channel. To confirm the feasibility of the two pathways, we computed the relevant diffusion energy barriers, respectively. Initially, we looked into the first case. Figure 7a depicts the detailed path of H_2_ diffusing along the macrocycle into the interior. The computed diffusion barriers are plotted in Figure 7b. It can be found that an energy of −9.91 eV is required for H_2_ to enter the tube’s interior through the macrocycle, which is too high to handle. In the second scenario, as displayed in Figure 7c, we conducted 1 × 1 × 2 cellular expansions of the C_3_N_4_ nanotube to diffuse H_2_ molecules from one Sc adsorption site to the neighboring Sc site and, subsequently, through the C_3_N_4_ tube channel along the z-axis. Figure 7d presents a plot of the estimated diffusion barriers. According to the findings, the H_2_ diffusion barriers are close to 0 eV, indicating that the H_2_ diffusion barrier is low along the tube channel of the C_3_N_4_ nanotube. As a result, we propose that H_2_ diffuses more readily along the channel than along the macrocycle in C_3_N_4_ nanotubes. Favorable conditions for H_2_ molecule transfer on Sc-modified C_3_N_4_ nanotubes are provided by a low diffusion barrier, which ensures efficient H_2_ molecule adsorption and desorption capacity.

### 2.6. Molecule Dynamics for H_2_ Desorption

Based on the Van Hove equation (Formula (5)), the obtained average desorption temperature is 258 K, lower than room temperature, which indicates that Sc-modified C_3_N_4_ nanotubes can release adsorbed hydrogen at near-ambient temperatures. The release temperatures for certain hydrogen storage substances are notably greater compared to ambient conditions [7,17]. This leads to a slow release rate in terms of adsorbed hydrogen at room temperature and cannot be used normally. Therefore, high-temperature treatments are applied to achieve the rapid desorption of hydrogen; however, high temperatures will destroy the structure of hydrogen storage materials. In contrast, Sc-modified C_3_N_4_ nanotubes can store hydrogen at low temperatures and release it at ambient temperatures for use, as shown in Figure S8. This will reduce energy consumption when facilitating the release of hydrogen, reaching about 10 kJ/mol H_2_ molecules compared with the case of some hydrogen storage materials containing MgH_2_ under standard conditions (referring to the section named “Energy Saving Compared with Some Hydrogen Storage Materials” in the Supplementary Materials). The low desorption temperature also protects the structural stability of the hydrogen storage system and increases its service life. Consequently, Sc-modified C_3_N_4_ nanotubes, boasting optimal average adsorption energy and release temperatures, have emerged as excellent candidates for fuel cell technologies.

## 3. Computation Details

The Device Studio software package (Version V2023A) [52] was employed to construct the computational models. DS-PAW software (Version V2023A) [53] was used to realize the simulation calculation of DFT, where Perdew–Burke–Ernzerhof (PBE) and generalized gradient approximation (GGA) [54] are adopted. The DFT has proven to be a reasonable calculation method and has been widely used to predict and verify hydrogen storage performance [55,56,57,58]. The DFT-D3 in the Grimme scheme was used to describe the van der Waals correction to simulate the various properties of C_3_N_4_ [59]. To reduce the interaction between C_3_N_4_ nanotubes, we placed C_3_N_4_ nanotubes in a 19 Å × 19 Å × 12 Å box for simulation. Since the vacuum spaces were all greater than 10 Å in the x-y plane, we can consider that there is almost no interaction between the mirror samples. In the process of structural optimization, to ensure the accuracy of the simulation, we set the cutoff energy to 450 eV, and set the convergence limits of force and energy to 0.05 eV/Å and 10^−4^ eV, respectively. To study the thermal stability of Sc-modified C_3_N_4_ nanotubes, we conducted AIMD simulations, where this system was maintained for 5ps at a temperature setting of 500 K.

The adsorption energy for a single H_2_ molecule $E_{b-H_{2}}$ on pure C_3_N_4_ nanotubes, indicating the change in energy during adsorption, is established as follows [60,61]:

(1) $$E_{b-H_{2}}=E_{total}-E_{H_{2}}-E_{sub}$$

where $E_{total}$, $E_{H_{2}}$, and $E_{sub}$ are the total energy of the adsorption system, isolated H_2_ molecule, and pure substrate (C_3_N_4_ or Sc-modified C_3_N_4_ nanotubes used in our study), respectively.

The bonding energies of a single Sc atom on pristine C_3_N_4_ can be obtained with the following formula:

(2) $$E_{b-S_{c}}=E_{S_{c}+C_{3}N_{4}}-E_{S_{c}}-E_{C_{3}N_{4}}$$

where $E_{S_{c}+C_{3}N_{4}}$, $E_{S_{c}}$, and $E_{C_{3}N_{4}}$ are the total energy of Sc-modified C_3_N_4_ nanotubes, isolated Sc atoms, and pure C_3_N_4_ nanotubes, respectively.

The mass fraction of Sc atom on C_3_N_4_ nanotubes can be obtained with the following formula:

(3) $$S_{c}-wt\%=\frac{m_{S_{c}}}{m_{S_{c}+C_{3}N_{4}}}\times 100\%$$

$S_{c}-wt\%$, $m_{S_{c}}\;$, and $m_{S_{c}+C_{3}N_{4}}$ denote the mass fraction of the Sc atom, the mass of Sc atoms, and the mass of the system of Sc + C_3_N_4_ nanotubes_._

The mass fraction of H_2_ on Sc-modified C_3_N_4_ nanotubes can be obtained with the following formula:

(4) $$H_{2}-wt\%=\frac{m_{H_{2}}}{m_{S_{c}+H_{2}+C_{3}N_{4}}}\times 100\%$$

$H_{2}-wt\%$, $m_{H_{2}}$, and $m_{S_{c}+H_{2}+C_{3}N_{4}}$ denote the mass fraction of the hydrogen, the mass of H_2_, and the mass of the system of H_2_ and Sc-modified C_3_N_4_, respectively.

To test the thermal stability and reversibility of the H_2_@Sc-modified C_3_N_4_ configuration in the practical application, the Van Hove equation was applied to estimate the average desorption temperature $T_{d}$, as follows [36]:

(5) $$T_{d}=(\frac{\bar{E_{b-H_{2}}}}{k_{B}})(\frac{\Delta S}{R}-lnP{)}^{-1}$$

where *R* denotes the gas constant, $k_{B}$ represents the Boltzmann constant, *P* is the atmospheric pressure, $\bar{E_{b-H_{2}}}$ signifies the mean adsorption energy for nine H_2_ atoms, which is approximately −0.20 eV/H_2_, and the change in entropy, represented by Δ*S*, which occurs during the transition of H_2_ from a gas to a liquid.

## 4. Conclusions

DFT simulations were performed to explore the possibility of storing hydrogen in pure C_3_N_4_ nanotubes. The results show that pure C_3_N_4_ nanotubes do not satisfy the requirements for storing H_2_; however, the addition of Sc to the nanotube allows for successful storage. According to the computed bonding energies and DOS, Sc atoms are stabilized in the macrocycle of C_3_N_4_, and covalent bonds are formed due to the fact that 1.83 electrons move to the N-2p states from the Sc-3d states. AIMD simulations and diffusion barriers confirm the structural stability of Sc-modified C_3_N_4_ nanotubes at high desorption temperatures. The diffusion barrier of Sc atoms from one macrocycle to its neighbor is 5.10 eV, which avoids the creation of metal clusters. From the perspective of hydrogen storage, up to nine hydrogen molecules can be absorbed on the Sc-modified C_3_N_4_ nanotube, with a hydrogen uptake of 7.29 wt%, which is above DOE-US requirements. The enhancement of the hydrogen storage capacity of Sc-modified C_3_N_4_ nanotubes is due to the charge donation and back donation from the Sc-3d to H-1s. Weak van der Waals and Kubas interactions are primarily responsible for this phenomenon. Additionally, the H_2_ diffusion route was investigated. According to the findings, H_2_ diffuses in C_3_N_4_ nanotubes more easily along the channel than along the macrocycle. Low-diffusion-barrier Sc-modified C_3_N_4_ nanotubes create favorable conditions for adsorption. The calculated average adsorption energy and desorption temperature are −0.20 eV and 258 K. For fuel cell applications, the Sc-modified C_3_N_4_ tube is suitable as it has appropriate average adsorption energy and desorption temperatures. We assert that Sc-modified C_3_N_4_ nanotubes are a promising and practically viable solution for high hydrogen storage.

## Data Availability

Data are contained within the article and Supplementary Materials.

## Supplementary Materials

The following supporting information can be downloaded at: https://www.mdpi.com/article/10.3390/molecules29091966/s1, Figure S1. Geometric structures of pure C_3_N_4_ nanotube and possible adsorption sites; Figure S2. The adsorption energy for one H_2_ molecule on different adsorption sites of pure C_3_N_4_ nanotube; Table S1. The bonding energy for Sc single atom on different deposition sites of pure C_3_N_4_ nanotube; Figure S3. Relative value of total energy variations as well as their corresponding fittings for the pristine (a) and Sc modified (b) C_3_N_4_ nanotubes with respect to strain ε along the tube axis; Figure S4. (a)–(f) The lowest-energy configuration of Sc modified C_3_N_4_ nanotube with the successive adsorption of 1 to 8 H_2_ molecules; Figure S5. The optimal structure of fully Sc modified C_3_N_4_ nanotube; Table S2. The effect of defects on the bonding energy and adsorption energy; Figure S6. The optimized configuration of Sc-modified C_3_N_4_ nanotubes adsorbing three H_2_O molecules; Figure S7. PDOS for H-1s orbital versus the Sc-3d orbital in C_3_N_4_+Sc+H_2_ systems. Fermi level is set at 0 eV; Figure S8. The application diagram of Sc-modified C_3_N_4_ nanotubes as a hydrogen storage material for storing, transporting, and releasing hydrogen [62,63,64,65,66].
