# Supplementary material for: Sc-Modified C3N4 Nanotubes for High-Capacity Hydrogen Storage: A Theoretical Prediction
Source: Molecules. 2024 Apr 25;29(9):1966. doi: 10.3390/molecules29091966 (PMC11085168; doi:10.3390/molecules29091966)
Supplement: Supplementary file 1 [file molecules-29-01966-s001.zip › molecules-2974011-supplementary.pdf]

Supporting Information

Sc-Modified C_3N_4 Nanotubes for High-Capacity Hydrogen Storage: A Theoretical Prediction

Shuli Liu [1], Xiao Tang [2], Chang He [1], Tingting Wang [1], Liying Shang [1], Mengyuan Wang [1], Shenbo Yang [3], Zhenjie Tang [1] and Lin Ju [1,*]

[1] School of Physics and Electric Engineering, Anyang Normal University, Anyang 455000, China; 01958@aynu.edu.cn (S.L.); 211104005@stu.aynu.edu.cn (C.H.); 211104029@stu.aynu.edu.cn (T.W.); 211104023@stu.aynu.edu.cn (L.S.); 211104027@stu.aynu.edu.cn (M.W.); zjtang@aynu.edu.cn (Z.T.)

[2] College of Science, Institute of Materials Physics and Chemistry, Nanjing Forestry University, Nanjing 210037, China; xiaotang@njfu.edu.cn

[3] Hongzhiwei Technology (Shanghai) Co., Ltd., 1599 Xinjinqiao Road, Pudong, Shanghai 201206, China; yangshenbo@hzwtech.com

* Correspondence: julin@aynu.edu.cn

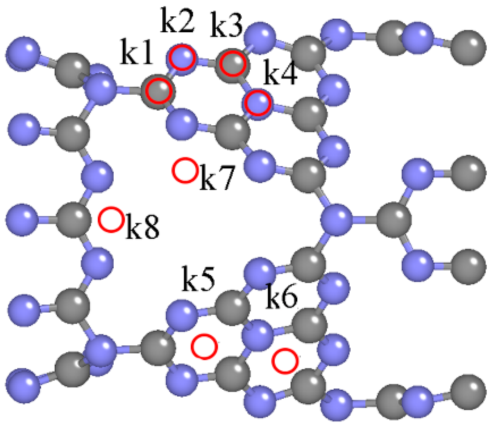

Figure S1. Geometric structures of pure C_3N_4 nanotube and possible adsorption sites.

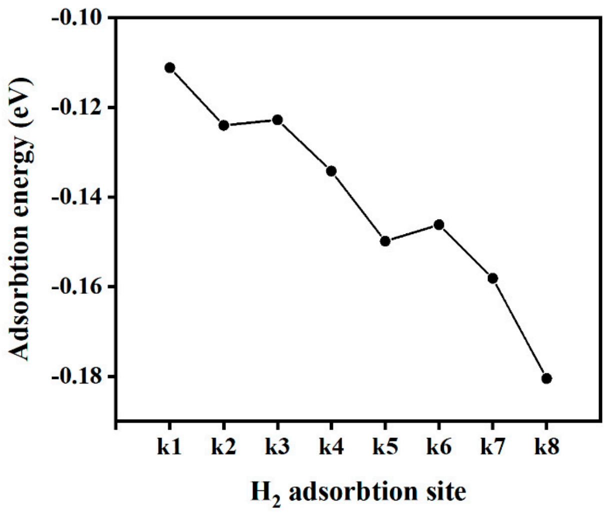

Figure S2. The adsorption energy for one H_2 molecule on different adsorption sites of pure C_3N_4 nanotube.

Table S1. The bonding energy of a single Sc atom on different deposition sites of a pure C_3N_4 nanotube.

Deposition sites (see Figure S1)	E_b (eV)
k1	-2.69
k2	-6.31
k3	-6.61
k4	-2.42
k5	-2.69
k6	-3.12
k7	move to the center (-6.62)
k8	move to the center (-6.62)

The Elastic Modulus of Pristine and Sc-modified C_3N_4 Nanotubes

The Young's modulus C of the 1D material can be described as follows [1],

$$C = \frac{1}{l_0}\left(\frac{\partial^2 E}{\partial \varepsilon^2}\right)$$

where l_0 is the lattice constant of a nanotube along the axial direction without strain; the strain ε is defined as $\varepsilon = \frac{l - l_0}{l_0}$, and E is the relative total energy of a nanotube under different strains (-0.03~0.03 along the axial direction), where the one without strain is treated as the standard value (seeing Figure S3). The calculated elastic modulus of pristine and Sc-modified C_3N_4 nanotubes is 241.81 and 232.13 eV Å^{-1}, respectively. That is to say, the elastic modulus of a C_3N_4 nanotube reduces by merely 4.00% after the introduction of a single Sc atom. Therefore, single-atom Sc decoration makes the C_3N_4 nanotube a bit softer.

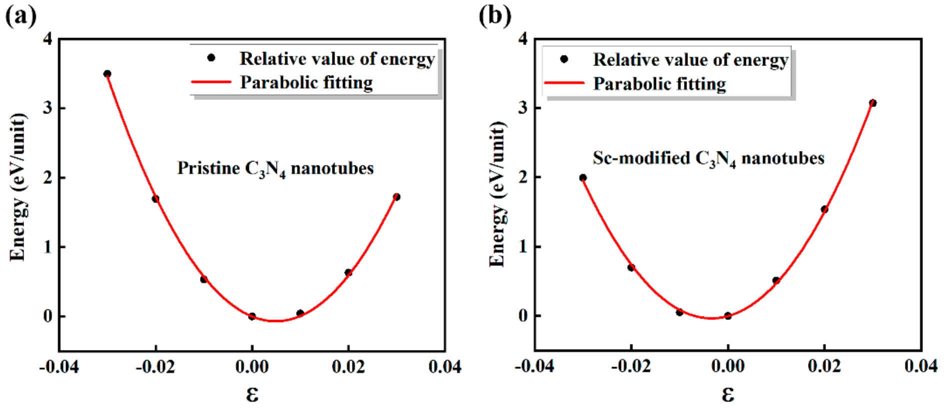

Figure S3. Relative value of total energy variations as well as their corresponding fittings for the pristine (a) and Sc-modified (b) C_3N_4 nanotubes with respect to strain ε along the tube axis.

The Effect of Exchange-Correlation Functional on the Migration Barrier of a Sc Atom

In order to explore the effect of the exchange-correlation functional on the migration barrier of a Sc atom, we changed the exchange-correlation functional to calculate the corresponding migration barrier. When the exchange-correlation functional was changed into PEBSOL and REVPEB, the calculated migration barrier was 6.21 eV and 5.31 eV, respectively. All of these methods show that a single Sc atom has a large migration barrier. In This work, PBE is used because it presents many advantages. The PBE functional has high accuracy when describing electron correlation, which can predict the physical properties of a material, such as the lattice parameter number and the density of the electron state. The PBE functional is suitable for a variety of material systems, including metals, semiconducting systems, insulators, and superconductors. The calculation cost of the PBE functional is relatively low, which is suitable for material calculations on a large scale.

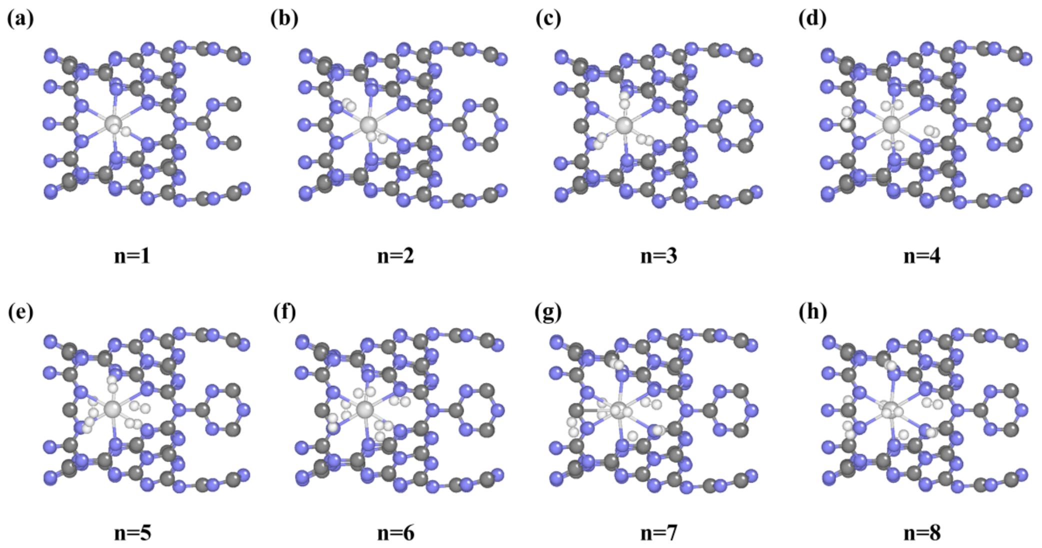

Figure S4. (a)~(f) The lowest-energy configuration of Sc-modified C_3N_4 nanotube with the successive adsorption of 1 to 8 H_2 molecules.

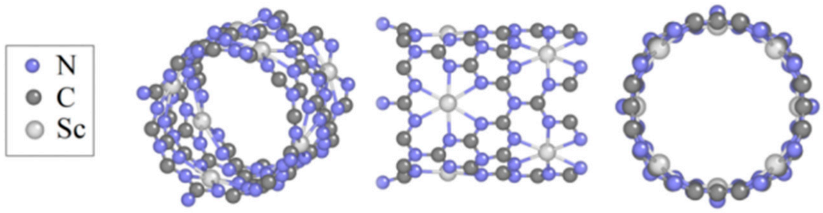

Figure S5. The optimal structure of fully Sc-modified C_3N_4 nanotube.

The Effect of Defects on Hydrogen Storage Efficiency and Stability

To further judge the effect of defects on the efficiency and stability of hydrogen storage, we established the Sc-modified C_3N_4 nanotube configuration with C and N defects, respectively, and calculated the bonding energy of a single Sc atom, as well as the adsorption energy of one hydrogen molecule. The obtained values are summarized in Table S2. The results show that with the introduction of C and N defects into a Sc-modified C_3N_4 nanotube, the bonding energy changes to -7.12 eV and -6.87 eV, respectively. Compared with the system without defects, the bonding energy decreases a little, which indicates that the introduction of defects could enhance the stability of Sc atom decoration in C_3N_4 nanotubes. However, when using the configuration with C/N defects adsorbing single H_2 molecules, the adsorption energy attenuated to -0.27 eV and -0.37 eV. The poor adsorption energy suggests that the introduction of defects reduces the efficiency and stability of hydrogen storage.

Table S2. The effect of defects on the bonding energy and adsorption energy.

Configuration	Bonding Energy	Adsorption energy
Perfect	-6.62 eV	-0.79 eV
C vacancy	-7.12 eV	-0.27 eV
N vacancy	-6.87 eV	-0.37 eV

The Effect of Humid Environments on Hydrogen Storage

To study the effect of humidity on the hydrogen storage of Sc-modified C_3N_4 nanotubes, we established a Sc-modified C_3N_4 configuration adsorbing three H_2O molecules to simulate a humid environment. The adsorption energy of the first hydrogen molecule is calculated to predict the effect of humidity on hydrogen adsorption. Through calculation, we find that the adsorption energy of this system is reduced to 0.073 eV. Such low energy cannot adsorb H_2. Through the optimized structure, it was found that a chemical bond formed between Sc atoms and two H_2O molecules (Fig. S5), which passives the active site of H_2 adsorption, thus hindering the adsorption of H_2 molecules. Therefore, to better realize the hydrogen storage of the Sc-modified C_3N_4 nanotube structure, we should perform hydrogen adsorption in a dry atmosphere.

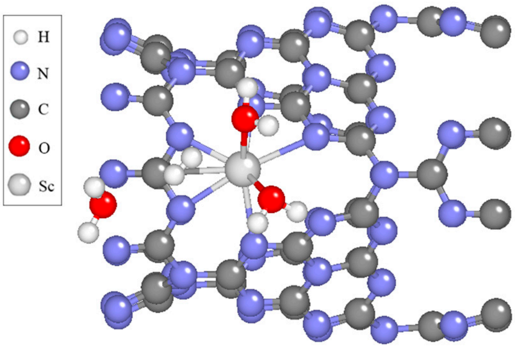

Figure S6. The optimized configuration of Sc-modified C_3N_4 nanotubes adsorbing three H_2O molecules.

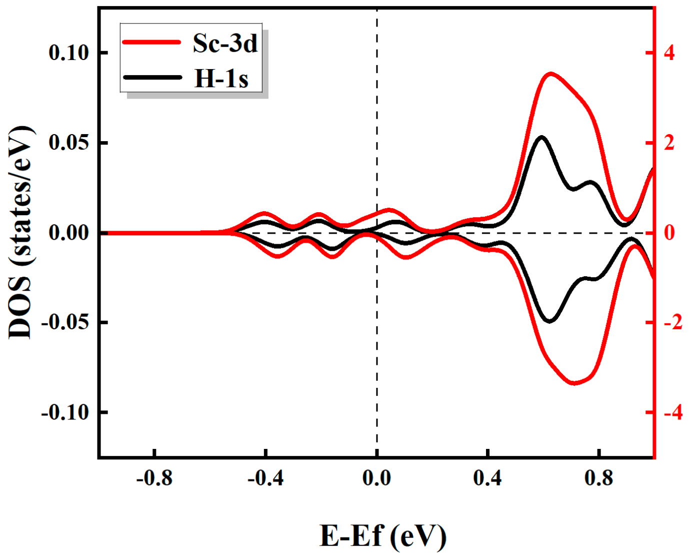

Figure S7. PDOS for H-1s orbital versus the Sc-3d orbital in C_3N_4+Sc+H_2 systems. Fermi level is set at 0 eV.

Storage Time for Hydrogen

Based on the analysis of the average adsorption energy of H_2, we find that Sc-modified C_3N_4 nanotubes can adsorb hydrogen at low temperatures and release it at ambient temperatures for use. As displayed in Fig. S8, in the process of storing hydrogen, we envision placing Sc-modified C_3N_4 nanotubes into a gas cylinder, then opening the valve of the hydrogen cylinder and placing the cylinder in a low-temperature hydrogen environment to allow it to spontaneously adsorb hydrogen. Once the hydrogen storage material reaches adsorption saturation, the valve of the gas cylinder is closed and transported at room temperature. Upon arrival to the scenario of use, the valve of the gas cylinder can be opened at room temperature to enable the Sc-modified C_3N_4 nanotube to release hydrogen. Therefore, as long as the seal of the hydrogen cylinder is sufficiently high, the storage time for hydrogen can be very long.

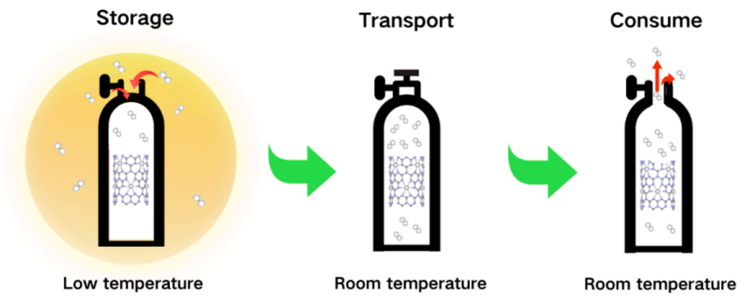

Figure S8. The application diagram of Sc-modified C_3N_4 nanotubes as a hydrogen storage material for storing, transporting, and releasing hydrogen.

Energy Saving Compared with Some Hydrogen Storage Materials

The Sc-modified C_3N_4 nanotubes can store hydrogen at low temperatures and release it at ambient temperatures for use. This will reduce energy consumption in facilitating the release of hydrogen. Compared with other hydrogen storage materials, the temperature of the released hydrogen is converted into energy, according to formula 5. The average desorption temperature of the Sc-modified C_3N_4 system is 258K, and the calculated average hydrogen release takes only 0.2 eV/H_2, and when compared to other hydrogen storage systems, our system is much more energy-efficient. For example, for MgH_2-5 wt% Ti_3C_2 (releasing hydrogen at a temperature of 458 K), the energy required to release hydrogen is 0.36 eV/H_2 [2]. The MgH_2-10 wt% $TiVO_{3.5}$ initiated hydrogen release at a temperature of 470 K, which is equal to 0.36 eV/H_2 [3]. Using a solid MXene$(Ti_{0.5}V_{0.5})_3C_2$ to reduce the starting temperature of hydrogen desorption of MgH_2 to 483 K is equal to 0.37 eV/H_2 [4]. Activated MgH_2-5 wt% $Nb_4C_3T_x$ starts to release hydrogen at 423 K, which is equal to 0.33 eV/H_2 [5]. Compared with these hydrogen storage materials, Sc-modified C_3N_4 nanotubes separately save 15.4, 15.4, 16.4, and 12.5 kJ/mol H_2 molecules under standard conditions.

Reference

1. Li, X.; Dai, Y.; Ma, Y.; Li, M.; Yu, L.; Huang, B. Landscape of DNA-like inorganic metal free double helical semiconductors and potential applications in photocatalytic water splitting. *J. Mater. Chem. A* **2017,** 5, 8484-8492.
2. Liu, Y.; Du, H.; Zhang, X.; Yang, Y.; Gao, M.; Pan, H. Superior catalytic activity derived from a two-dimensional Ti_3C_2 precursor towards the hydrogen storage reaction of magnesium hydride. *Chem. Commun.* **2016,** 52, 705.
3. Zhang, X.; Shen, Z.; Jian, N.; Hu, J.; Du, F.; Yao, J.; Gao, M.; Liu, Y.; Pan, H. A novel complex oxide $TiVO_{3.5}$ as a highly active catalytic precursor for improving the hydrogen storage properties of MgH_2. *Int. J. Hydrogen Energ.* **2018,** 43, 23327-23335.
4. Shen, Z.; Wang, Z.; Zhang, M.; Gao, M.; Hu, J.; Du, F.; Liu, Y.; Pan, H. A novel solid-solution MXene $(Ti_{0.5}V_{0.5})_3C_2$ with high catalytic activity for hydrogen storage in MgH_2. *Materialia* **2018,** 1, 114-120.
5. Liu, Y.; Gao, H.; Zhu, Y.; Li, S.; Zhang, J.; Li, L. Excellent catalytic activity of a two-dimensional $Nb_4C_3T_x$ (MXene) on hydrogen storage of MgH_2. *Appl. Surf. Sci.* **2019,** 493, 431-440.
